# Rosmarinic Acid Prevents Cisplatin-Induced Liver and Kidney Injury by Inhibiting Inflammatory Responses and Enhancing Total Antioxidant Capacity, Thereby Activating the Nrf2 Signaling Pathway

**DOI:** 10.3390/molecules27227815

**Published:** 2022-11-13

**Authors:** Yifei Xiang, Min Ji, Liqin Wu, Li Lv, Qiuling Liang, Ruihan Deng, Zhaoyou Deng, Xia Liu, Lingyi Ren, Xin Feng, Jiakang He

**Affiliations:** 1 College of Animal Science and Technology, Guangxi University, Nanning 530005, China; 2 Department of Pharmaceutics and Drug Delivery, School of Pharmacy, University of Mississippi, Oxford, MS 38677, USA

**Keywords:** acute liver and kidney injury, rosmarinic acid, cisplatin, inflammation, oxidative stress, Nrf2

## Abstract

Drug-induced liver and kidney damage is an emergent clinical issue that should be addressed. Rosmarinic acid (RA) has obvious anti-inflammatory and antioxidant effects, so we evaluated the anti-inflammatory and antioxidant effects of RA pretreatment on serum and liver and kidney tissues of cisplatin (CP)-treated mice and explored the possible mechanisms. The results showed that RA pretreatment effectively downregulated the serum, liver, and kidney levels of ALT, AST, BUN, and CRE and the inflammatory factors IL-1β, IL-6, and TNF-α, and simultaneously enhanced the total antioxidant capacity of the liver and kidney. RA pretreatment significantly reduced the levels of MPO, MDA, and NO in liver and kidney tissue, inhibited the mRNA expression of IL-1β, IL-6, and TNF-α in liver and kidney tissue, activated the Nrf2 signaling pathway, and upregulated the mRNA expression of downstream target genes. Our findings show that RA could effectively prevent and alleviate acute liver and kidney injury caused by CP.

## 1. Introduction

Drug-induced liver and kidney damage is caused by toxic reactions to a drug and its toxic metabolites, excessive use of drugs, traditional Chinese herbal medicines, or dietary supplements, and acute liver and kidney injury (ALI and AKI) are common syndromes caused by medical side effects. To date, the number of deaths due to AKI and ALI has exceeded one million [1,2], and, therefore, is a clinically urgent medical problem and hotspot issue in the medical community [3,4]. According to incomplete statistics, at least 1100 drugs currently have liver and kidney toxicity, which is also the main reason why some drugs are capped in the market, withdrawn from the market, or do not receive approval [5,6].

Cisplatin (CP) is one of the earliest and most widely used chemotherapeutic drugs in clinical practice. It is often used to treat various tumor diseases in the body [7], but severe liver and kidney toxicity limit its application in clinical treatment. A large number of studies have shown that CP can induce oxidative stress and an inflammatory response in the body, and the positive effects of the two can lead to liver and kidney damage [8,9].

Nuclear factor-erythroid 2-related factor 2 (Nrf2) is a transcription factor that plays an important regulatory role in oxidative stress and inflammatory responses [7]. Nrf2 is an important transcription factor that we have been paying attention to and is widely found in various organs of the body. Under physiological conditions, Nrf2 can combine with Keap1 in the cytoplasm so that Nrf2 is at a reduced level, thereby maintaining the body in a homeostatic environment by regulating antioxidant capacity and inflammatory factors. The liver and kidney toxicity of CP promotes the activation of inflammatory factors and accelerates the production of ROS (reactive oxygen species), thereby inducing oxidative stress in the body. The oxidative stress response accelerates the release of Nrf2 from Keap1, and high levels of Nrf2 disrupt the homeostasis of the body, thereby accelerating the occurrence of liver and kidney damage. Some literature points out that amifostine has been approved by the FDA for the treatment of nephrotoxicity caused by CP, but it has a high cost and many side effects [6]. Therefore, it is necessary to develop a drug that can be used to alleviate and treat the liver and kidney toxicity caused by CP.

Rosmarinic acid (RA) is a polyphenolic derivative obtained from Perilla species with various pharmacological activities, such as antioxidant, anti-inflammatory, antitumor, liver, and kidney protection activities [10]. Studies have shown that RA alleviates damage mainly by inhibiting the activation of inflammatory and oxidative stress-related pathways [11,12,13]. Previously, we reported that RA alleviated ALI and allergic asthma in mice by inhibiting inflammatory and oxidative stress responses [14,15,16,17]. However, whether RA, as an activator of Nrf2, can prevent CP-ALI and AKI through this pathway is unclear. Here, we explore and reveal that RA alleviates drug-induced acute liver and kidney injury by activating the Nrf2 signaling pathway to bidirectionally regulate oxidative stress and the inflammatory response and provide a pharmacodynamic theoretical basis for the development of RA as a clinical drug for alleviating liver and kidney injury.

## 2. Results

### 2.1. Effects of RA on Body Weight Changes and Organ Indices in Mice with CP-ALI and AKI

**Figure 1:**
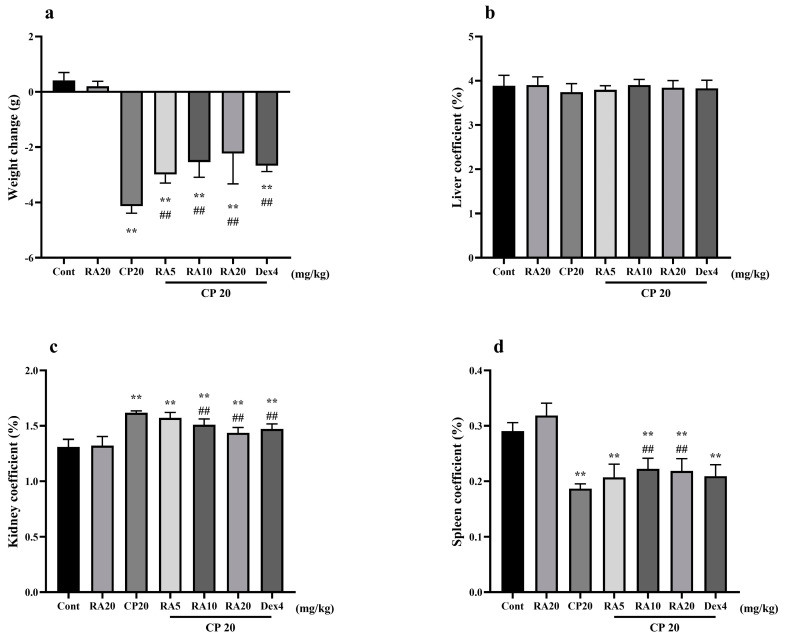
Effects of RA on body weight changes and organ indices in mice with CP−induced ALI and AKI. The body weight, liver, kidney, and spleen of mice were weighed, their body weight changes (**a**) are presented, and their liver coefficients (**b**), kidney coefficients (**c**), and spleen coefficients (**d**) were calculated. These experiments were repeated three times. ** and ## denote highly significant differences (*p* < 0.01) compared with the control and CP model groups, respectively.

The body weight of mice in the control group and those given RA alone gradually increased over time (*p* > 0.05), whereas mice given CP to induce injury both showed a significant decrease in body weight compared with the control group (*p* < 0.01). Compared with mice in the CP model group, body weight loss became slower in the two groups given RA and dexamethasone (DEX) pretreatment (*p* < 0.01), and the difference in body weight between these two groups was not significant (*p* > 0.05), with the best effect in alleviating body weight loss in mice being the one given 20 mg/kg BW (body weight) RA (Figure 1a).

There were no significant changes in the liver indices of mice in each group (*p* > 0.05) (Figure 1b). Compared with that in the control group, the kidney index in the CP model group was significantly increased (*p* < 0.01), and the spleen index in the RA control group was slightly increased (Figure 1c,d). This effect is helpful for immunity and is worthy of further research in our follow-up experiments. After drug prophylaxis and challenge with CP, the kidney indices in the RA (10, 20 mg/kg BW) and DEX-pretreatment groups were significantly decreased (*p* < 0.01), whereas the spleen indices were significantly increased (*p* < 0.01). The results showed that RA (20 mg/kg BW) pretreatment and the control drug DEX had similar effects on alleviating visceral injury in mice.

### 2.2. Effects of RA on Liver and Kidney Function in Mice with CP-ALI and AKI

**Figure 2:**
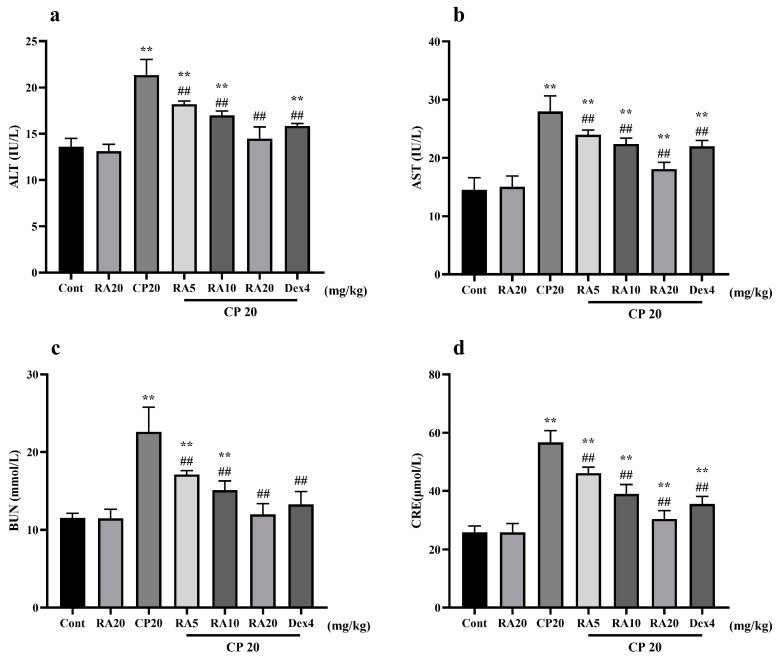
The effect of RA on the liver and kidney function indices ALT, AST, BUN, and CRE in CP-induced ALI mice. The expression levels of the liver function indices AST and ALT (**a**,**b**) and the kidney function indices BUN and CRE (**c**,**d**) were detected by ELISA. These experiments were repeated three times. ** and ## denote highly significant differences (*p* < 0.01) compared with the control and CP model groups, respectively.

In the determination of liver and kidney functions in mice, it was found that the expression levels of serum ALT, AST, BUN, and CRE in the group administered with CP alone did not differ significantly from those in the control group, whereas the model group given CP showed significant differences (*p* < 0.01) (Figure 2). The liver and kidney indices of mice pretreated with RA and DEX showed a significant downward trend compared with the CP model group (*p* < 0.01), and interestingly, the preventive effect of giving 20 mg/kg BW RA was even better than that of DEX. These results showed that RA pretreatment had a protective effect against liver and kidney injury caused by CP, especially 20 mg/kg BW RA.

### 2.3. Effect of RA on Serum Levels of IL-1β, IL-6 and TNF-α Induced by CP

**Figure 3:**
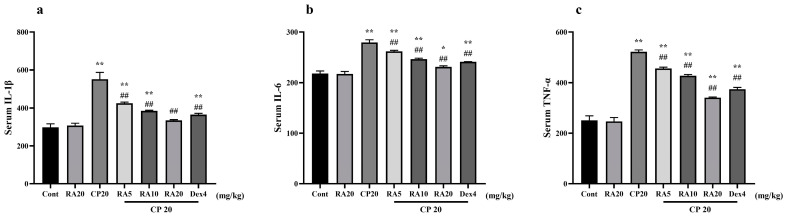
Effects of RA on CP-induced levels of IL-1β, IL-6, and TNF-α in serum. The expression levels of the serum inflammatory factors IL-1β (**a**), IL-6 (**b**) and TNF-α (**c**) in mice were detected by ELISA. These experiments were repeated three times. * denote significant differences (*p* < 0.05) and ** and ## denote highly significant differences (*p* < 0.01) compared with the control and CP model groups, respectively.

Detection of serum IL-1β, IL-6 and TNF-α inflammatory factors in different groups of mice revealed that the expression levels of serum inflammatory factors were significantly higher (*p* < 0.01) in mice in the CP model group and control group (Figure 3). In contrast, the mice given pretreatment with RA and DEX showed significantly lower serum inflammatory-factor expression levels compared with the CP model group (*p* < 0.01), and the effect of pretreatment with 20 mg/kg BW RA was more similar to the efficacy of DEX. The results showed that the high level of inflammation caused by CP could be well suppressed after administration of RA pretreatment, and the best performance was observed when 20 mg/kg BW was administered.

### 2.4. Effects of RA on the Antioxidant Capacity of the Liver and Kidney in the Presence of CP

**Figure 4:**
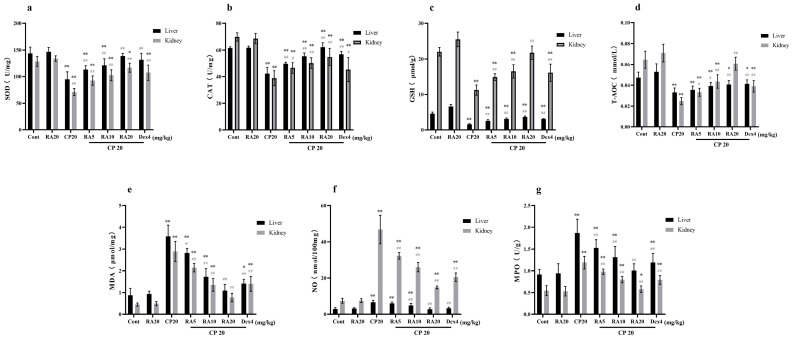
The effect of RA on the expression levels of SOD, CAT, GSH, T-AOC, MDA, NO, and MPO in the liver and kidney of mice induced by CP. The liver and kidney tissues were homogenized, the supernatant was taken, and the antioxidant capacity of the liver and kidney tissues was detected by ELISA (**a**–**g**). These experiments were repeated three times. * and # denote significant differences (*p* < 0.05) and ** and ## denote highly significant differences (*p* < 0.01) compared with the control and CP model groups, respectively.

After measuring the antioxidant capacity of liver and kidney tissues, it was found that the administration of RA alone did not significantly alter the differences in the indices when observed in comparison with those of the control group, but the administration of CP significantly decreased the antioxidant capacity of liver and kidney tissues (*p* < 0.01), with the expression of SOD, CAT, GSH, and T-AOC being downregulated (Figure 4a–d), and the activities of NO and MPO were most significantly upregulated (Figure 4e–g). Compared to the decrease in antioxidant capacity caused by CP, both RA and DEX administration significantly reversed this trend (*p* < 0.01). The final results showed that the administration of RA pretreatment improved the antioxidant capacity of the mouse liver and kidney and showed a dose-dependent effect, with the administration of 20 mg/kg BW RA showing the most outstanding antioxidant capacity in combination, which was even better than the control drug DEX.

### 2.5. Effects of RA on mRNA Expression in CP-Induced ALI and AKI

**Figure 5:**
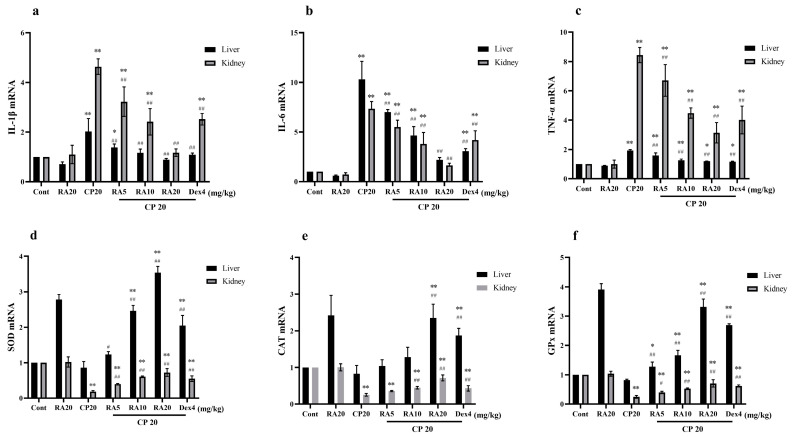
The effect of RA on the mRNA expression levels of IL-1β, IL-6, TNF-α, SOD, CAT, and GPx in CP-induced acute liver and kidney injury. The mRNA expression levels of IL-1β, IL-6, TNF-α, SOD, CAT, and GPx in the liver and kidney of mice were measured by qRT–PCR (**a**–**f**). These experiments were repeated three times. * and # denote significant differences (*p* < 0.05) and ** and ## denote highly significant differences (*p* < 0.01) compared with the control and CP model groups, respectively.

Compared with the control group, IL-1β, IL-6, and TNF-α mRNA expression levels in liver and kidney showed a significant upregulation (*p* < 0.01) and renal SOD, CAT, and GPx mRNA expression levels showed a significant downregulation (*p* < 0.01) after CP induction administration (Figure 5). After RA administration alone, liver SOD, CAT, and GPx mRNA expression levels showed abnormal upregulation, and the remaining indices were not significantly different from the control group. After administration of the RA pretreatment, hepatic and renal IL-1β, IL-6, and TNF-α mRNA expression levels showed a significant downregulation (*p* < 0.01) and SOD, CAT, and GPx mRNA expression levels showed a highly significant upregulation (*p* < 0.01) compared to CP induction alone.

These results suggest that administration of RA pretreatment reverses CP-induced inflammatory changes and improves the mRNA expression of SOD, CAT, and GPx, and the antioxidant capacity of liver and kidney tissues. Taken together, administration of 20 mg/kg BW RA had the best anti-inflammatory and antioxidant effects, and even the preventive effect bi preventive effect was superior to the control drug.

### 2.6. Effects of RA on Histological Changes of CP-Induced ALI and AKI in Mice

**Figure 6:**
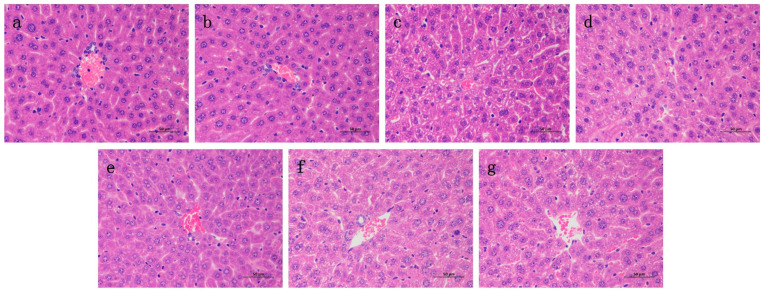
Histological observation of RA on CP-induced ALI in mice and the differences between groups were observed using H&E staining at 400× magnification. Pathological examination of control group (**a**) mice; (**b**) optimal therapeutic dose of RA (20 mg/kg) given to mice; (**c**) CP (20 mg/kg) challenged mice; (**d**–**f**) RA (5, 10, 20 mg/kg) treated CP-challenged mice; (**g**) mice were challenged with CP of the DEX (4 mg/kg) control treatment drug.

**Figure 7:**
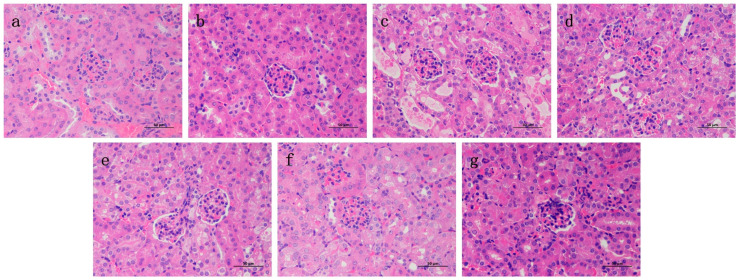
Histological observation of RA on CP-induced AKI in mice and the differences between groups were observed using H&E staining at 400× magnification. Pathological examination of control group (**a**) mice; (**b**) optimal therapeutic dose of RA (20 mg/kg) given to mice; (**c**) CP (20 mg/kg) challenged mice; (**d**–**f**) RA (5, 10, 20 mg/kg) treated CP-challenged mice; (**g**) mice were challenged with CP of DEX (4 mg/kg) control treatment drug.

Liver and kidney histology in the RA control drug group was similar to that in the control group (Figure 6 and Figure 7), and there were no obvious histopathological changes. The liver tissue in the CP model group showed slight local vacuolar degeneration of hepatocytes, disordered arrangement of renal tissue, degeneration of the proximal tubules, a brush border disappearance, faded cytoplasmic staining, nuclear pyknosis, fragmentation or dissolution, and renal tubular epithelial cell degeneration. RA pretreatment alleviated the damage caused by CP to varying degrees, and 20 mg/kg BW RA had the most obvious preventive effect, resulting in the lowest degree of liver and kidney tissue damage.

### 2.7. Effects of RA on CP-Induced mRNA Expression Levels of the Nrf2 Signaling Pathway

**Figure 8:**
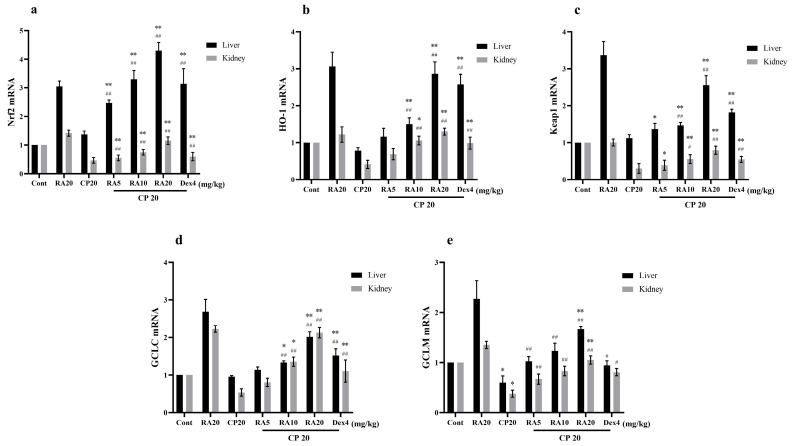
Effects of RA on the expression levels of Nrf2 signaling pathway-related target genes in CP-induced liver and kidney tissues. The mRNA expression levels of Nrf2 (**a**), HO-1 (**b**), Keap1 (**c**), GCLC (**d**), and GCLM (**e**) in the liver and kidney of mice were detected by qRT–PCR. These experiments were repeated three times. * and # denote significant differences (*p* < 0.05) and ** and ## denote highly significant differences (*p* < 0.01) compared with the control and CP model groups, respectively.

Compared with the blank group, the hepatic GCLM mRNA expression level after CP induction was significantly downregulated (*p* < 0.05); Nrf2, HO-1, Keap1, and GCLC mRNA showed a downregulation trend but not significantly (*p* > 0.05), whereas the renal Nrf2, HO-1, Keap1, GCLC, and GCLM mRNA expression levels showed a significant downregulation trend (*p* < 0.01) (Figure 8). Compared with the CP model group, administration of RA and DEX pretreatment significantly reversed the downregulation of Nrf2-associated factors (*p* < 0.01). The results suggest that administration of the RA pretreatment attenuates the CP damage to the liver and kidney.

## 3. Discussion

CP is a platinum-based broad-spectrum anticancer drug that is commonly used in the treatment of various tumor diseases [18,19], but dose-dependent liver and kidney toxicity greatly limits the clinical application and efficacy of CP [20,21,22]. Therefore, the use of CP-induced BALB/c mice AKI and ALI pathological models to study the related problems of the disease has been the focus of our attention. According to the previous research of the research group, we finally established a pathological model of acute liver and kidney injury via a single intraperitoneal injection of 20 mg/kg BW CP [23]. The occurrence of ALI and AKI is strongly associated with oxidative stress and inflammatory responses, and the development of a drug that inhibits inflammation and oxidative stress responses is an alternate approach to explore for the treatment of drug-induced liver and kidney injury.

The Nrf2 signaling pathway plays an important role in the occurrence and development of acute liver and kidney injury. When the body is stimulated by external substances, it will automatically activate the Nrf2 signaling pathway to resist oxidative stress and inflammatory responses [24,25,26]. Nrf2 is a major regulator of oxidative stress and inflammatory responses in liver and kidney tissue cells against foreign toxic substances. Under physiological conditions, Nrf2 and Keap1 are coupled in the cytoplasm. After oxidative stress occurs, Nrf2 and Keap1 rapidly dissociate, enter the nucleus, and combine with ARE, thereby regulating the gene expression of related antioxidants, such as SOD, CAT, GPx, HO-1, GCLC, and GCLM. Among them, HO-1 is an important detoxification enzyme in the body. Recent studies have shown that it has antioxidant and anti-inflammatory effects. The side effects of drugs cause the body to generate too much ROS, and a large amount of ROS will damage RNA, protein, various macromolecules in tissues and cells, and damage the antioxidant capacity of liver and kidney tissues [27], making the body’s own oxidation and antioxidation. These antioxidant enzymes can work together to remove excess ROS in liver and kidney tissues and inhibit oxidative stress and inflammatory responses to alleviate liver and kidney tissue damage [28,29]. Oxidative stress and inflammation have a complex relationship, and oxidative stress can accelerate the activation of inflammation-related signaling pathways, including NF-κB [7]. When the body is stimulated by drug side effects, macrophages and neutrophils release many cytokines and inflammatory mediators related to inflammation, including IL-1β, IL-6, TNF-α, and chemokines. Excessive production of cytokines and other mediators can lead to necrosis or apoptosis of liver and kidney cells, resulting in aggravated liver and kidney damage or even loss of function.

RA is a traditional plant polyphenol derivative of ongoing interest to us, and previous studies have shown it to be safe and non-animal toxic [30]. Our previous study has demonstrated that RA can protect mice from LPS-induced ALI [14]. In another study, RA was shown to effectively downregulate inflammatory factor levels in the lung by limiting NF-κB and MAPK signaling pathway-related mRNA expression in lung tissues, thus providing a protective effect in a mouse model of asthma [15]. In addition, RA was shown to inhibit MAPK phosphorylation and activate the NF-κB signaling pathway, effectively delaying airway inflammation and providing a potential therapeutic option for the treatment of allergic asthma [16]. For the first time, it was demonstrated that RA pretreatment could effectively alleviate oxidative stress and inflammatory damage in mouse lung tissue, thereby preventing and treating allergic asthma [17]. Previous explorations of RA have shown that RA has an effect on oxidative stress and inflammation, but whether it can challenge cisplatin-induced drug-induced liver and kidney damage by limiting inflammation and oxidative stress responses, and thus through the Nrf2 signaling pathway, remains unclear. Thus, we hypothesized that RA could prevent CP-induced ALI and AKI, thereby opening new avenues for exploring drug-induced liver and kidney injury. Xiaoye Fan [7] used Daphnin to enhance the antitumor activity of cisplatin and reduce its induced nephrotoxicity by activating the nrf2 pathway. In another study, he used isoprene rubber to activate the Nrf2 pathway, thereby inhibiting oxidative stress and apoptosis to reduce cisplatin-induced nephrotoxicity [31]. Jianqiang Hu [32] further attenuated drug-induced kidney injury by activating the Nrf2 signaling pathway induced by CP using motherwort.

Dexamethasone (DEX) is a synthetic glucocorticoid that has been shown to have anti-inflammatory, antioxidant, antiviral, anti-shock, and immunosuppressive pharmacological effects in multiple studies [33]. The study by Daniela Gabbia [34] showed that low-doses of DEX can alleviate liver dysfunction and its accompanying oxidative stress and inflammatory responses. Zihe Zhai [35] included DEX with nanomaterials, which showed very good antioxidant capacity and inflammation inhibitory capacity. More studies have used DEX as a control drug to compare the anti-inflammatory and antioxidant capacity of the compound on the body [36]. To evaluate the protective effect of RA on ALI and AKI in mice with CP, we referred to previous experience and selected DEX as a preventive reference drug.

Because the CP-induced mouse model of ALI and AKI has a high degree of clinical similarity with the occurrence and development of the disease in humans, and because the model is simple and easy to replicate, easy to induce, inexpensive, and widely applicable [37], we used the CP-induced BALB/c mouse AKI and ALI model to study the related issues of these diseases, which is our long-term focus. According to our previous study, a single intraperitoneal injection of 20 mg/kg BW CP could be used to establish a pathological model of ALI and AKI. Our results showed that CP-induced mice lost body weight, had an abnormal visceral index, and that serum, liver, and kidney function levels were significantly higher than normal levels, and further, that serum inflammatory factor expression levels were significantly increased and that severe oxidative stress reactions occurred in liver and kidney tissues. Tissue lesions treated with RA prophylaxis in mice can reverse the above situation.

In conclusion, this study demonstrated that RA can dose-dependently control related inflammatory factors and antioxidant enzymes, and upregulate the expression levels of HO-1, Keap1, GCLC, and GCLM, thereby activating the Nrf2 signaling pathway to combat CP-induced oxidative stress in the body and inflammatory response-induced liver and kidney damage.

## 4. Materials and Methods

### 4.1. Animals

We purchased 70 6-week-old male BALB/c mice (SPF grade), weight control 18–22 g, from Hunan Slaughter Jingda Laboratory Animal Co., Ltd (Hunan, China). With the animal license number SCXK (Xiang) 2016-0002. In order to advocate the principle of animal welfare, our feeding method simulates the original growth environment of mice as much as possible, and the test was conducted after 7 days of adaptation. The experiments were conducted under the approval and support of the Animal Research Ethics Committee of Guangxi University, and all animal experiments were conducted according to the Guide for the Care and Use of Laboratory Animals published by the National Institutes of Health.

### 4.2. Reagents

BUN, CRE, ALT, AST, CAT, GSH, T-AOC, NO, and MPO test kits were purchased from the Jiancheng Institute of Biological Engineering (Nanjing, China), and mouse IL-1β, IL-6, and TNF-α ELISA kits were purchased from Xinbosheng Biotechnology Co., Ltd. (Shenzhen, China), and SOD and MDA detection kits were purchased from Beyotime Biotechnology (Nanjing, China). RNA Keeper Tissue Stabilizer, RNA Isolater Total RNA Extraction Reagent, HiScript III RT SuperMix for qPCR (+gDNA wiper), and ChamQ Universal SYBR qPCR Master Mix were obtained from Novezan Biotechnology Co., Ltd. (Nanjing, Jiangsu, China). CP was purchased from Sigma (Shanghai, China), and RA (HPLC ≥ 98%, batch number: 19080601) was purchased from Pfeide Biotechnology Co., Ltd. (Chengdu, Sichuan, China), and DEX (HPLC ≥ 98%, batch number: 100129-201506) was purchased from the China Institute for Food and Drug Control (Beijing, China).

### 4.3. Challenge and Treatment of Mice

Mice were randomly divided into 7 groups (10 mice per group): control group, RA simple administration group (20 mg/kg), CP model group (20 mg/kg), RA pretreatment group (5, 10, 20 mg/kg), and DEX pretreatment group (4 mg/kg). The pretreatment group continued with the corresponding dose of drug for 5 d. The blank and CP model groups were given an equal volume of 0.9% NaCl at an injection volume according to 0.1 mL/10 g body weight during the same period, and these doses were referred to our previous studies [15,38]. On the 5th day after treatment, mice, except the control group and the RA-only administration group, were induced with a single intraperitoneal injection of CP at 20 mg/kg BW to induce their liver and kidney injury.

### 4.4. Collection and Testing of Blood and Organs

The body weight of the mice was carefully observed and recorded during the test, blood was collected from the eyes on the 8th day of the test with the help of ether, centrifuged at 3000 rpm for 10 min at 4 °C after 2 h of standing, the serum was collected, and the liver and kidney function and inflammation levels of the mice were evaluated according to the instructions.

The liver, spleen, and kidney were carefully removed, washed with saline and blotted on filter paper, and then weighed and recorded. Tissues used for the determination of inflammatory factors and antioxidant capacity should be homogenized and stored as soon as possible after removal.

### 4.5. Real-Time Polymerase Chain Reaction (qPCR)

RNA was extracted from tissues with TRIzol (Novezan Biotechnology Co., Ltd., Nanjing, Jiangsu, China) according to the instructions and 1 μg of RNA was reverse-transcribed to cDNA using a reverse transcription kit. 20 μL of the reaction mixture was amplified by qRT–PCR using the reaction procedure in the ChamQ Universal SYBR qPCR Master Mix instructions (primers sequences are listed in Table 1). The relative expression levels of target genes in liver and kidney tissues were calculated according to the 2-ΔΔCt method.

### 4.6. Liver and Kidney Histology

The tissues were washed with saline solution and then fully immersed in 4% fixative for 48 h. The paraffin-embedded sections were stained with H&E and observed.

### 4.7. Statistical Analysis

The experimental data were analyzed by one-way ANOVA using GraphPad Prism 9 software (GraphPad Software, San Diego, USA) and expressed as mean ± standard deviation. * indicates compared with control group, # indicates compared with CP model group, * and # indicate significant difference (*p* < 0.05), ** and ## indicate highly significant difference (*p* < 0.01).

## 5. Conclusions

In this study, the protective effects of RA pretreatment on CP-induced ALI and AKI in mice were investigated for the first time through in vivo experiments, and it was confirmed that RA could inhibit inflammatory responses and antioxidant levels, which in turn activated genes related to the Nrf2 signaling pathway and reversed drug-induced hepatic and renal injury. These findings suggest that RA has potent preventive and protective effects on CP-induced hepatic and renal injury, thus opening a new avenue for exploring drug-induced hepatic and renal injury.

## Figures and Tables

**Table 1:** Primer Sets for Reverse Transcriptase-Polymerase Chain Reaction Analysis.

Targets	Forward	Reverse
IL-1β	AAAAAAGCCTCGTGCTGTCG	GTCGTTGCTTGGTTCTCCTTG
IL-6	CTAGTGCGTTATGCCTAAGC	ATAGTGTCCCAACATTCATATTGTC
TNF-α	AGCACAGAAAGCATGATCCG	CTGATGAGAGGGAGGCCATT
SOD	CGGCTTCTCGTCTTGCT	GTTCACCGCTTGCCTTC
CAT	GCCAACTACCAGCGTGA	CCGCACCTGAGTGACAT
GPx	GGCATTGGCTTGGTGATT	AGGTGGAAAGGCATCGG
Nrf2	GCTCCTATGCGTGAATCCCA	TTCTGGGCGGCGACTTTATT
HO-1	GTCAAGCACAGGGTGACAGA	AAGTGACGCCATCTGTGAGG
Keap1	ACCTCGGACTCGCAGCGTAC	CCAAGCAGGAGGAGTTCTTCAACC
GCLC	CTGCACATCTACCACGCAGT	GTCTCAAGAACATCGCCTCC
GCLM	CGGGAACCTGCTCAACTG	CCAAAACATCTGGAAACTCCC
β-actin	ATCACTATTGGCAACGAGCG	TCAGCAATGCCTGGGTACAT

## Data Availability

Not applicable.
